# The Value of Caspase-3 after the Application of *Annona muricata* Leaf Extract in COLO-205 Colorectal Cancer Cell Line

**DOI:** 10.1155/2017/4357165

**Published:** 2017-04-09

**Authors:** Murdani Abdullah, Ari Fahrial Syam, Sofy Meilany, Bayu Laksono, Oryza Gryagus Prabu, Heri Setiyo Bekti, Lili Indrawati, Dadang Makmun

**Affiliations:** ^1^Division of Gastroenterology, Department of Internal Medicine, Faculty of Medicine, Dr. Cipto Mangunkusumo Hospital, Universitas Indonesia, Jakarta, Indonesia; ^2^Institute of Human Virology and Cancer Biology, Faculty of Medicine, Dr. Cipto Mangunkusumo Hospital, Universitas Indonesia, Jakarta, Indonesia; ^3^Department of Pharmacotherapy, Faculty of Medicine, Christian University of Indonesia, Jakarta, Indonesia

## Abstract

*Annona muricata*, commonly known as soursop, contains annonacin, acetogenin, and polyphenol which are known to have chemopreventive effects on cancer. In this study, we tend to evaluate the apoptosis-inducing effect of soursop (*Annona muricata*) leaf extract on the colorectal cancer cell line COLO-205 through the activities of caspase-3 which is a marker of cell apoptosis. Cell cultures were incubated with soursop leaf with a concentration of 10 *μ*g/ml and then compared with those of the incubated positive control leucovorin 10 *μ*g/ml and placebo as a negative control. The apoptotic activity of caspase-3 was measured with ELISA. After the administration of each treatment in the colorectal cancer cell line COLO-205, the expression of caspase-3 activity was 1422 ng/ml after incubation with the extract of *Annona muricata* leaves, 1373 ng/ml after the administration of leucovorin, and 1297 ng/ml in the one with placebo. *Annona muricata* leaf extract elevated caspase-3 by 1.09 times compared to that of the pure cell line. *Annona muricata* leaf extract had a higher value of caspase-3 activity than leucovorin and placebo in the COLO-205 colorectal cancer cell line. These results may suggest that *Annona muricata* leaf extract had anticancer properties by enhancing caspase-3 activity which is a proapoptotic marker.

## 1. Introduction

Cancer is the second leading cause of death in developing countries. These cancer cases are found in developing countries partly due to the diverse populations of age, lifestyle, and environmental factors such as smoking, low-physical activity, Western food, and exposure to variety of jobs [1, 2]. The prevalence of colorectal cancer is increasing in Asia [3]. Colorectal cancer lined up in the third rank of cancers among men and women. Based on the International Cancer Research data, the incidence of colorectal cancer in Asia is similar to that seen in Europe. In the last decade in East Asia such as China, Japan, South Korea, and Singapore, cases of colorectal cancer have increased to two to four times [4]. In Indonesia, colorectal cancer was recently considered one of the most common cancers. From the 13 types of cancers in the registry data, colorectal cancer is one of the five most common cancers found in men and women [5].

There has been approaches using supportive therapy as adjuvant for chemical therapy such as chemotherapy. This supportive therapy can be taken from natural products [6, 7]. Natural product derivatives have flavonoid, terpenoid, and steroid which include cytotoxic and chemopreventive effects [8, 9]. These effects can inhibit tumor initiation, promotion, and progression. Therefore, the scientific validation of traditional medicine should be done for its possible use in the prevention and treatment of cancer. To fight cancer cells, there is an apoptotic pathway need to be taken by the natural product [6]. Caspase is one of the apoptotic pathways in order to perform cell death. There is overwhelming experimental evidence supporting the idea that inducing caspase response can help cell cancer to become apoptotic [10].

Classification of human caspase is based on their function, size of their prodomain, and cleavage activity. The function of inflammatory caspase is represented by group I caspase, and they are caspases 1, 4, and 5 that are involved in cytokine maturations. Regulations of apoptosis is controlled by group II caspase. There are two classes of group II caspase, and they are the initiator caspases which are performed by caspases 2, 8, 9, and 10 and the effector caspases which are performed by caspases 3, 6, and 7. Effector caspases are produced in cells as dimer and proteolytic processing by an initiator enzyme to trigger its activity. After being active, the effector can target cells leading to program the apoptosis [10].

Caspase-3 is one of the effector caspases that can be measured for the number of apoptosis in cancer cells. Caspase-3 can be initiated by either extrinsic factor or intrinsic factor. Extrinsic factor will initiate FasL and lead to the cleavage of FasR. This leads to the separation of the large and small subunits of catalytic domain. Afterwards, it will activate caspase 8 and induce the activation of caspase-3 which leads to the apoptosis of cells [11].

Nowadays, there are increasing research on natural products to find anticancer activity. One of the anticancer activities can be found from herbal. Herbal with acetogenin activity can be found in the leaves of *Annona muricata* [12, 13]. They contain acetogenins which are known to have cytotoxic effect in tumor cells. Polyphenol activity from *Annona muricata* leaves has chemopreventive effect by lowering the incidence of some types of cancer, especially in colonic epithelial cells [9, 14, 15].

## 2. Materials and Methods

### 2.1. Plant Material and Extraction

The material used in this experiment is the soursop leaf extract. The leaves were extracted with a litre of 96% ethanol at room temperature by the Indonesian Institute of Sciences. This results in 1/20 ethanol-soluble material (we called it EIFAM extract). The ethanol-soluble extract was dried until all alcohol contents evaporated (we called it ESFAM extract). For positive control, we used certified standardized material products produced by *Javaplant* which are soluble materials (we called them ORAC extract). We also used 5-fluorouracil and leucovorin for positive control.

### 2.2. Cell Culture

Cell line used in this study was COLO-205 cell line. COLO-205 was derived from colorectal adenocarcinoma tissues of Dukes of type D with poor differentiation in men aged 70 years from ATCC [16]. COLO cells were grown using DMEM media with 10% FBS, 100 *μ*/ml antibiotic penicillin streptomycin, and 2 mM L-glutamin. Cells were grown in 75 cm^3^ and placed in an incubator with 5% CO_2_ concentration and 95% humidity. Before the treated cells were maintained for 3 days until cells reach 90%, cells were then trypsinized using 0.25% trypsin EDTA. Cells were then centrifugated at 1000 rpm for 5 minutes to separate pellets from supernatant. Supernatant was discharged and replaced with new complete media to neutralize the effect of trypsin EDTA.

### 2.3. Cytotoxicity Assay

The cytotoxicity test was performed using colorimetric assay, MTT assay 3-(4,5-dimethythiazol-2-yl)-2,5-diphenyl tetrazolium bromide. The cells were seeded in a 96-well plate with a density of 1 × 10^5^/well using DMEM complete media which contain 10% FBS and 100 *μ*g/ml penicillin streptomycin. After it reaches the confluency, the media were then replaced with another media which contain ORAC extract, EIFAM extract, ESFAM extract, 5-flourouracil, and leucovorin with concentration of 400 ppm, 200 ppm, 100 ppm, 50 ppm, 25 ppm, 12.5 ppm, 6.25 ppm, 3125 ppm, and 15,625 ppm and were then incubated for 24 and 48 h. At the end of each time point, 10 *μ*l of 5 mg/ml MTT [3-(4,5-dimethylthiazol-2-yl)-2,5-diphenyltetrazolium bromide] solution was added (final concentration 0.5 mg/ml and stock solution 5 mg/ml MTT in PBS) for 4 h. The MTT solution and medium were removed and 100 *μ*l DMSO was added to each well. Absorbance was measured at 570 nm using the ELISA microplate reader.

### 2.4. Measurement of Caspase-3 Activity

We calculated caspase-3 using ELISA method obtained by R&D System. The plates were conjugated with recombinant caspase-3 and provided with monoclonal antibody raised against caspase-3. All samples, standards, and reagents are performed at room temperature then added to each well for 100 *μ*l each and were incubated at room temperature for 2 hours. After the incubation, each well was aspirated and washed 5 times. The wells were then added with conjugate, 100 *μ*l for each well, and then were incubated at room temperature for 1 hour and proceeded with washing the plates 5 times. Then, the plates were added with substrate solution of 100 *μ*l and were incubated at room temperature for 30 minutes. Stop solution was added to each well and read the plates at 450 nm with correction wavelength of 540 nm. Caspase-3 was available in ng/ml which means the higher value was the better value of caspase-3.

## 3. Results

### 3.1. Cell Viability and Cytotoxicity

**Figure 1:**
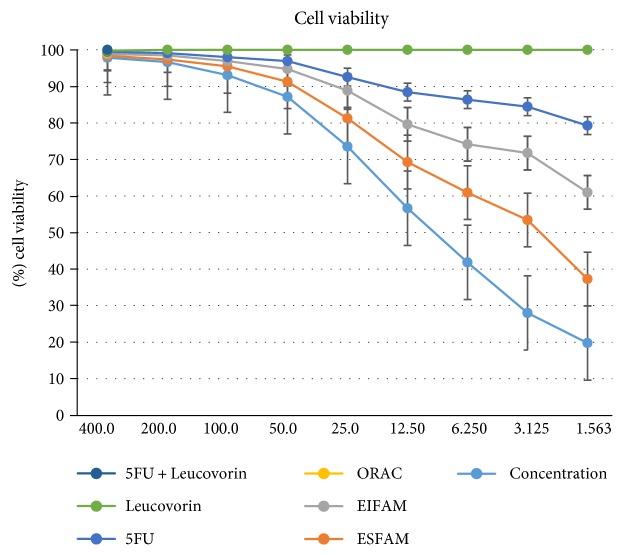
COLO-205 cell lines were treated with ESFAM, EIFAM, leucovorin, and 5FU for 48 h. Cell viability was determined using MTT assay. The results represent the mean ± SD of three independent experiments.

MTT assay was performed to evaluate the effect of EIFAM, ESFAM, ORAC, 5FU, leucovorin, and a combination of 5FU and leucovorin (FULV) on the survival of COLO-205 cells (Figure 1). A number of living cells were found in percentage. All extracts were performed three times. From the result, we found that ESFAM has good results compared to the other extracts (Table 1).

From the result, we found that after 48 hours of incubation, cell viability of *Annona muricata* leaf extract was 21.01% whereas leucovorin cell viability was 201.85%. The cell viability was reduced to 61.3% after incubated with *Annona muricata* leaf extract for 48 hours. However, lower cell viability was observed in *Annona muricata* leaf extract compared to that in leucovorin with a significant statistical value (*p* < 0.05). In 48 hours of incubation, the IC-50 of *Annona muricata* leaf extract and leucovorin was 189.6 *μ*g/ml and 87.41 *μ*g/ml, respectively (data not shown).

#### 3.1.1. Caspase-3

**Figure 2:**
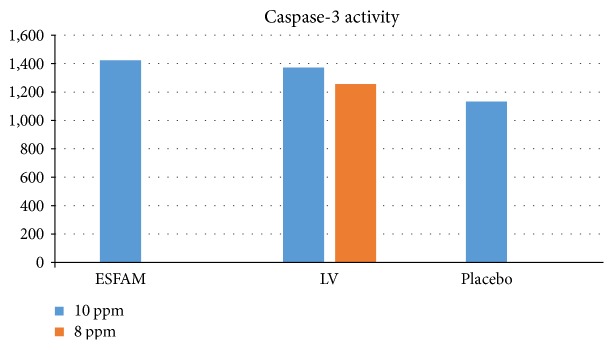
Caspase-3 activity of ESFAM, leucovorin, and placebo with 10 ppm and 8 ppm dosages. ESFAM extract gives a higher result at 10 ppm dosage compared to that at 8 ppm dosage.

For detection of caspase-3 activity, we compared two dosages of each extract and we used 10 *μ*g/ml and 8 *μ*g/ml of dosage for ESFAM, leucovorin, and placebo. From the ELISA result, we found that the ESFAM extract with a concentration of 10 *μ*g/ml gives higher result of caspase, which gives a concentration value of 1422 ng/ml than that of leucovorin and placebo where the values of caspase-3 were 1373 ng/ml and 1297 ng/ml, respectively (Figure 2). *Annona muricata* leaf extract elevated caspase-3 by 1.09 times.

## 4. Discussion

Increasing incidence of colorectal cancer worldwide needs to be balanced with the development of treatments both chemical and phytochemical [3, 6]. Soursop (*Annona muricata*) leaves contain acetogenins, alkaloids, flavonoids, terpenoids, coumarins, and so on. These components could be found either in a form of water or in ethanol extracts, but in different levels of concentration. Flavonoid level is high in the form of ethanol extracts and low in water. There are a number acetogenins contained in Annonaceae which has anticancer effects [17, 18]. The leaves of *Annona muricata* contain the highest number of flavonols compared to the roots or twigs [19]. The natural component of *flavonolol quercetin* is found mainly in plants and has an anticancer effect [20].

Caspase-3 is the biomarker which is used as a parameter in this study. Caspase-3 is a protease that is most often associated with cell death which catalyzes many cellular proteins and mediates the *α*-fodrin, gelsolin, and ICAD/DFF-45 which alter the morphology of nuclear and the process of apoptosis [21].

The cytotoxic value of *Annona muricata* leaf extract obtained in this study showed good results and significance based on time which can inhibit cell proliferation up to 45% in 48 hours after the treatment is being given. Through this research, the amount of IC-50 of *Annona muricata* leaf extract in colorectal cancer cell culture COLO-205 was observed. IC-50 is the amount of concentration of *Annona muricata* leaf extract required in inhibiting 50% cell proliferation in vitro. The smaller the number required, the more potent the drug was [22].

The research conducted by Zorofchian Moghadamtousi et al. demonstrated that *Annomuricin E* from *Annona muricata* leaves inhibits the growth of HT-29 cells with IC-50 in the number of 1.62 ± 0.24 *μ*g/ml after 48 hours [23]. In another study by Pieme et al., it was found that the IC-50 of *Annona muricata* inhibits the growth of HL-60 cells (leukemia) in the range of 6–12 *μ*g/ml after 48 hours [19]. This shows that a different number of dosage is required in a different type of cancer. Higher concentration of IC-50 was needed in this study compared to that in another study. Different types and different fruits from each country may be different in the concentration of active components that have proapoptotic agent. However, in this study, the value of the active components for proapoptotic function was not measured. In the study conducted by Fidaningsih and Handayani, it is found that the best effect of the water extract of soursop leaves to inhibit proliferation is in the dosage of 75 mg/ml with an 88.45% inhibition rate [24]. On the other hand, in another study by Astirin et al., it is found that the dosage needed for alcohol-soluble soursop leaf extract to achieve the same effect is 2 mg/ml with 35.80% apoptotic figure [25]. Another study which assessed the effect of the leaves of *Annona muricata* against cancer cells described that the leaf extract could induce apoptosis in colon and lung cancer through the mitochondria and has the effect of activation of caspase-3 in leukemia. It is also stated that the anti-inflammatory effect is achieved by lowering the migration of leukocytes and the volume of exudate [26]. In a study conducted by Niu et al., it is found that there was a 1.1 times increase in caspase-3 in COLO-205 which was mediated through mitochondria [20]. However, Mondal et al. found no effects of elevation of caspase-3 with the intervention of *Annona* [27].The same result was obtained by Yuan et al. who stated that there was a decrease of activity of procaspase-3 after 8 hours of administration of annonacin, a type of acetogenin [28].

In this study, *Annona muricata* leaf extract had a 1.09 times higher caspase-3 activity compared to the placebo and 1.03 times higher compared to the leucovorin. Leucovorin acts as precursor for 5,10-methylenetetrahydrofolate, which is needed to form the ternary complex with FdUMP (contained in 5-flourouracil) and thymidylate synthase (TS, which is an important target of chemotherapy), is essential for long-term maintenance of TS inhibition. Leucovorin effect can also increase the availability of CH2-THF which will be polyglutamylated and will trigger the inhibition of TS. With the absence of CH2-THF or one of the polyglutamates, FdUMP forms an unstable binary complex, which results in poor inhibition [29, 30].

On the other side, most acetogenin annonacea especially *Annona muricata* was defined by between one and three THF rings with one or two hydroxyl groups on the long-chain hydrocarbon [31]. *Annona muricata* has an adjacent bis-tetrahydrofuran ring system which has the most powerful potential cytotoxicity [32, 33]. This result could suggest the role of *Annona muricata* as combination therapy such as leucovorin.

## 5. Conclusion

*Annona muricata* leaf extract had a better value of caspase-3 compared to leucovorin and placebo. These results may suggest that *Annona muricata* leaf extract had anticancer properties by enhancing the caspase-3 activity which is a proapoptotic marker. However, further research to determine which of the active fraction of *Annona muricata* leaves has the effect of increasing the activity of caspase-3 is of great demand. It will enrich the scientific evidence of *Annona muricata* leaf activity as a proapoptotic agent for cancer.

## Figures and Tables

**Table 1:** Result of cell viability assay using MTT assay in percentage. This showed that ESFAM extract has the best result compared to others.

	Cell viability (%)
ESFAM	*61.30*
EIFAM	114.21
FU	83.15
Leucovorin	100.71
